# Pericarditis in Takotsubo Cardiomyopathy: A Case Report and Review of the Literature

**DOI:** 10.1155/2013/917851

**Published:** 2013-12-17

**Authors:** Joonseok Kim, Heather S. Laird-Fick, Osama Alsara, Venu Gourineni, George S. Abela

**Affiliations:** Department of Medicine, Michigan State University, East Lansing, MI 48824, USA

## Abstract

*Case*. A 64-year-old Caucasian woman was brought to the emergency department with severe dysphagia and left chest pain for last 4 days. Initial evaluation revealed elevated ST segment in precordial leads on EKG with elevated cardiac enzymes. Limited echocardiogram showed infra-apical wall hypokinesia. Cardiac angiography was done subsequently which showed nonflow limiting mild coronary artery disease. Takotsubo cardiomyopathy was diagnosed and she was treated medically. On the third day of admission, a repeat ECG showed diffuse convex ST-segment elevations in precordial leads, compatible with acute pericarditis pattern of EKG. Decision was made to start colchicine empirically for possible pericarditis. Follow-up EKG in 2 days showed decreased ST-segment elevations in precordial leads. The patient was discharged with colchicine and a follow-up echocardiogram in 4 weeks demonstrated a normal ejection fraction with no evidence of pericarditis. *Conclusion*. Acute pericarditis can be associated either as a consequence of or as a triggering factor for Takotsubo cardiomyopathy. It is vital for physicians to be aware of pericarditis as a potential complication of Takotsubo cardiomyopathy.

## 1. Introduction

Takotsubo cardiomyopathy (TC), also known as transient apical ballooning syndrome or broken heart syndrome, is a sudden onset nonischemic cardiomyopathy that causes akinesis of the left midventricle and apex. Patients usually present with acute chest pain and ST segment elevation on ECG that mimics an acute coronary syndrome. The prognosis of TC is mostly favorable. However, serious complications can occur including cardiogenic shock, ventricular rupture, dysrhythmia, and pericarditis [1]. The relationship between TC and pericarditis has not been clearly identified. Here we report a case of Takotsubo cardiomyopathy that was possibly complicated with asymptomatic pericarditis.

## 2. Case Presentation

A 64-year-old Caucasian woman was brought to the emergency department after being found confused. She had been feeling nauseated and was unable to keep anything down due to severe dysphagia for the last 4 days. She also reported that she had been having left side chest pain for the last 3-4 days, and it became much worse on the day of presentation. She was a long-term smoker and had a history of hypertension, dyslipidemia, insulin-dependent diabetes, and depression.

**Figure 1:**
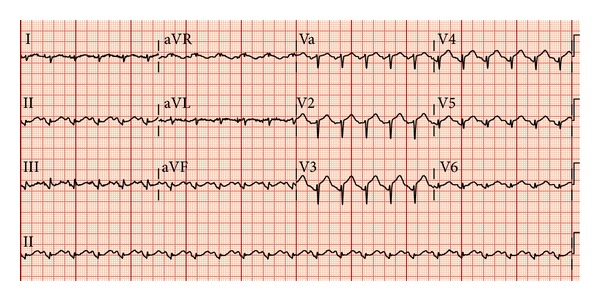
Initial ECG on presentation showing ST-segment elevation in leads V2 to V4, II, III, and aVF.

**Figure 2:**
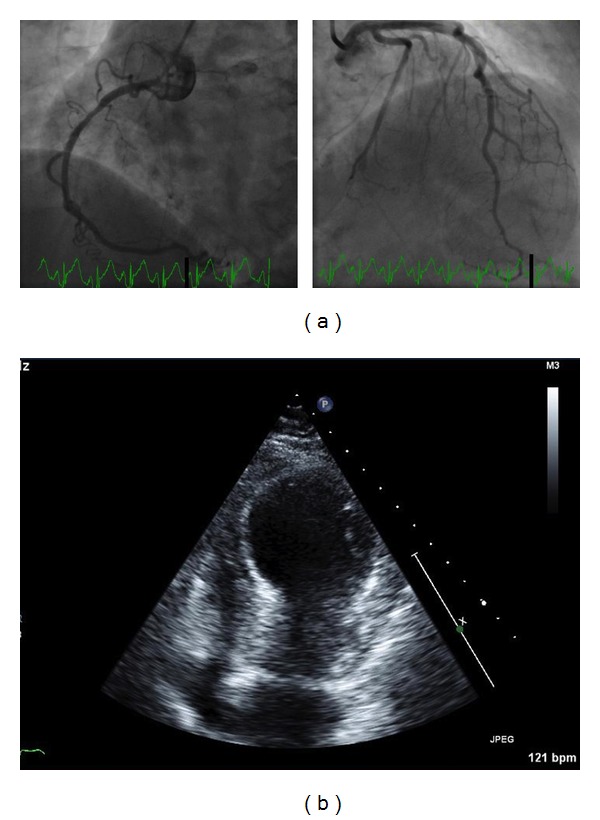
(a) Coronary angiography showing nonobstructing 30% stenosis of right coronary artery with otherwise normal coronary arteries. (b) Transthoracic echocardiogram showing anteroapical and infra-apical wall hypokinesis with ejection fraction of 20%.

The initial electrocardiogram (ECG) revealed a normal sinus rhythm and ST-segment elevation in leads V2 to V4, II, III, and aVF (Figure 1). The bedside echocardiogram showed anterior wall hypokinesis. Her initial laboratory work-up reported a WBC count of 26000, and urine analysis showed a urinary tract infection. The first set of cardiac enzyme tests was positive with a troponin level of 23.61 ng/mL (normal range <0.02), CPK of 1092 U/L (normal range <155 U/L), and CK-MB fraction total value of 41.5 ng/mL (normal range <6.3 ng/mL). She was given aspirin, nitroglycerin, and a beta-blocker and her chest pain diminished. The patient was immediately transferred to the cardiac catheterization laboratory for primary coronary intervention of a possible ST-elevation myocardial infarction (STEMI). Cardiac angiography demonstrated a proximal 30% nonobstructive tubular stenosis of the right coronary artery, but otherwise normal coronary arteries (Figure 2(a)). Anteroapical and infra-apical wall hypokinesis with an ejection fraction of 20% was noted on repeat echocardiogram (Figure 2(b)). Takotsubo cardiomyopathy was diagnosed based on the clinical presentation and coronary angiographic findings. The beta-blocker and low-dose aspirin were continued.

The next morning, her chest pain, and dysphagia subsided substantially. Cardiac enzymes that peaked with a troponin of 32.66 ng, CPK of 1211 U/L, and CK-MB fraction total value of 47.00 ng/mL were trending down. Antibiotics to treat sepsis secondary to the urinary tract infection were continued and the WBC count came down to 19300. Esophagogastroduodenoscopy (EGD) was done to evaluate her dysphagia, which showed normal findings other than a mild gastritis.

**Figure 3:**
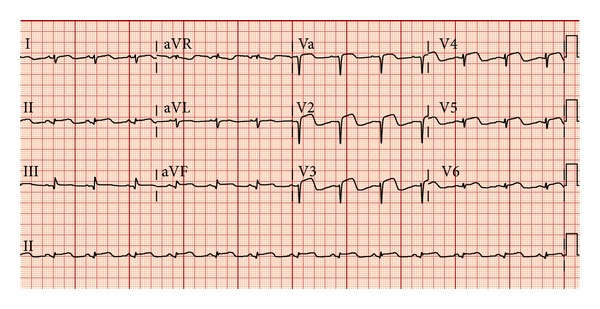
ECG on the third day of admission shows diffuse convex ST-segment elevations in leads V1 to V6, II, III, and aVF.

On the third day of admission, a repeat ECG showed new diffuse convex ST-segment elevations in leads V1 to V6, II, III, and aVF (Figure 3). The patient denied dyspnea, palpitation, or chest pain with respiration. Physical examination revealed a normal sinus rhythm and no pericardial friction rub or murmurs. Erythrocyte sedimentation rate was >100 mm/hour and C-reactive protein was <1.0 mg/dL, which was most likely from acute inflammation and pericarditis than sepsis or UTI. The decision was made to start colchicine empirically for possible pericarditis. NSAIDs were avoided due to the patient's gastric discomfort. She was discharged on hospital day 7 with colchicine and a beta-blocker. The colchicine was continued to complete the 10-day course of empirical treatment. A follow-up echocardiogram done 4 weeks after discharge demonstrated a normal ejection fraction and no wall-motion abnormality.

## 3. Discussion

Takotsubo cardiomyopathy (TC) is a type of nonischemic cardiomyopathy that is characterized by sudden onset chest pain and elevated ST segments on ECG. Approximately 1 to 2% of all suspected acute myocardial infarctions turn out to be Takotsubo cardiomyopathy [10].

The first Takotsubo-like reversible cardiomyopathy was described in Japan in 1990 [11]. Since then, the number of the diagnoses of TC has increased rapidly [12]. TC is a relatively benign cardiomyopathy and most of the patients' heart functions recover to normal in 1 to 3 months [9]. However, significant complications including cardiogenic shock, ventricular rupture, and dysrhythmia can occur as a consequence of TC even though they are rare [1].

As in the case of our patient, pericarditis can be associated either as a consequence of or as a triggering factor for TC [13]. The Takotsubo-pericarditis association has been identified in 8 previous case reports (Table 1). According to the reports, 7 out of 8 patients were female and the mean age was 76.2 after excluding one pediatric patient who was 14 years of age. TC preceded pericarditis in three cases but the time difference varied (3 days to 7 weeks). In four other cases, the patients were suspected of having acute pericarditis based on the presenting symptoms and initial ECGs but were later found to have Takotsubo pericarditis.

Although several case reports have identified the possible TC-pericarditis association, the exact pathogenesis remains unclear. Several theories have been hypothesized to explicate the TC-pericarditis connection. First, the transmural inflammation or myocarditis associated with TC is thought to have extended to the pericardium resulting in pericardial inflammation [4]. Nef et al. and other researchers demonstrated an infiltration of mononuclear inflammatory cells within the endomysium in patients with TC [14–16]. Avegliano et al. verified possible localized inflammation and edema in the affected area of TC by early cardiac magnetic resonance with late gadolinium enhancement imaging [17]. These study findings imply that an inflammatory process is associated with TC, which can potentially extend to the adjunct pericardium.

Another theory maintains that viral myocarditis could mimic TC and potentially spread to the pericardium [13]. There are several case reports in which biopsy-proven viral myocarditis presents with left ventricular apical ballooning. Studies have shown that approximately 15% of acute pericarditis is associated with underlying myocarditis or myopericarditis [18]. This suggests that cases originally thought to be TC may have actually been myocarditis, mimicking TC [19, 20]. Lastly, acute pericarditis could be the primary event and TC could be its consequence [13]. Pericarditis can cause severe pain, which could prompt catecholamine release [13]. Studies have shown that TC patients have significantly higher levels of catecholamines compared to those with myocardial infarction [16].

In conclusion, TC can potentially be associated with pericarditis. In the case of our patient, we believe that asymptomatic pericarditis was associated as a consequence of TC. Recognition of the TC and pericarditis association is significant because pericarditis itself can cause serious sequelae including cardiac tamponade and constrictive pericarditis if untreated. Furthermore, it would be hazardous if TC patients with pericarditis were unnecessarily exposed to thrombolytic therapy. A case of cardiac tamponade secondary to intravenous heparin and glycoprotein 2b/3a inhibitor in a patient with TC has been reported [5]. Therefore, it is vital for physicians to be aware of pericarditis as a potential complication of TC. Further study should be warranted to identify the pathophysiology of TC and its relationship to pericarditis.

## Figures and Tables

**Table 1:** Reported cases with regards to TC and pericarditis association.

Authors	Year	Age/sex	Preceding events	Following events	Time difference	Treatment	Outcome
Guevara et al. [2]	2007	84/F	Takotsubo cardiomyopathy	Cardiogenic shock, pericarditis	9 days	NSAID	Dramatic improvement (chest pain resolved)

Lee et al. [3]	2008	75/F	Takotsubo cardiomyopathy	Pericarditis and pericardial effusion	7 weeks	NSAID	Chest pain resolved

Maruyama et al. [4]	2007	65/F	Takotsubo cardiomyopathy	Pericarditis	3 days	NSAID	Chest pain resolved

Yeh et al. [5]	2010	83/F	Takotsubo cardiomyopathy	Cardiac tamponade	Immediately after	Pericardiocentesis	Follow-up echocardiogram 2 weeks after discharge shows normal EF and wall motion

Lee et al. [6]	2010	14/M	Presented as pericarditis, found to have Takotsubo cardiomyopathy			Pericardiocentesis	Gradually recovered. MRI at 14 days after the initial symptoms shows normal LV function and normal coronary arteries

Cambronero et al. [7]	2010	74/F	Pericarditis suspected initially based on typical ECG and symptom. Echo and subsequent ECG reveal Takotsubo cardiomyopathy				Discharged on the 9th day with normal EF

Li et al. [8]	2010	67/F	Pericarditis suspected initially based on typical ECG. Echo reveals Takotsubo cardiomyopathy			NSAID	Follow-up echocardiogram 4 weeks after discharge shows large pericardial effusion with cardiac tamponade. Pericardial window formation was done and patient recovers in a week

Jimmy and Foo [9]	2011	86/F	Pericarditis suspected initially based on typical ECG. Echo reveals Takotsubo cardiomyopathy				Not mentioned
